# Daubenton’s bats maintain stereotypical echolocation behaviour and a lombard response during target interception in light

**DOI:** 10.1186/s40850-024-00200-4

**Published:** 2024-04-29

**Authors:** Astrid Saermark Uebel, Michael Bjerre Pedersen, Kristian Beedholm, Laura Stidsholt, Marie Rosenkjaer Skalshøi, Ilias Foskolos, Peter Teglberg Madsen

**Affiliations:** 1 Section for Zoophysiology, Department of Biology, Aarhus University, Aarhus, Denmark; 2 Department of Evolutionary Ecology, Leibniz Institute for Zoo and Wildlife Research, Berlin, Germany; 3 Section for Wildlife Ecology, Department of Ecoscience, Aarhus University, Aarhus, Denmark

**Keywords:** Biosonar, Noise effect, Lombard response, Active sensing, Vision, Multimodality, Subcortical reflex

## Abstract

Most bats hunt insects on the wing at night using echolocation as their primary sensory modality, but nevertheless maintain complex eye anatomy and functional vision. This raises the question of how and when insectivorous bats use vision during their largely nocturnal lifestyle. Here, we test the hypothesis that the small insectivorous bat, *Myotis daubentonii*, relies less on echolocation, or dispenses with it entirely, as visual cues become available during challenging acoustic noise conditions. We trained five wild-caught bats to land on a spherical target in both silence and when exposed to broad-band noise to decrease echo detectability, while light conditions were manipulated in both spectrum and intensity. We show that during noise exposure, the bats were almost three times more likely to use multiple attempts to solve the task compared to in silent controls. Furthermore, the bats exhibited a Lombard response of 0.18 dB/dB_noise_ and decreased call intervals earlier in their flight during masking noise exposures compared to in silent controls. Importantly, however, these adjustments in movement and echolocation behaviour did not differ between light and dark control treatments showing that small insectivorous bats maintain the same echolocation behaviour when provided with visual cues under challenging conditions for echolocation. We therefore conclude that bat echolocation is a hard-wired sensory system with stereotyped compensation strategies to both target range and masking noise (i.e. Lombard response) irrespective of light conditions. In contrast, the adjustments of call intervals and movement strategies during noise exposure varied substantially between individuals indicating a degree of flexibility that likely requires higher order processing and perhaps vocal learning.

**Supplementary Information:**

The online version contains supplementary material available at 10.1186/s40850-024-00200-4.

## Introduction

Animals obtain critical information from their environment by relying on a range of different sensory systems, where information is gathered through either passive or active sensing [1]. A prime example of active sensing is the echolocation of bats who probe their surroundings via emission of powerful ultrasonic calls and subsequent auditory processing of weak returning echoes [2]. Masking noise from surroundings may accordingly compromise the detectability of these echoes and thereby complicate the echolocation process [3]. Like most vertebrates, bats can mitigate the effects of masking noise by increasing call source levels via the so-called Lombard response [4, 5], which partially defends echo-to-noise ratios [6]. Additionally, bats can enable sensory redundancy in returning echoes by either decreasing call intervals [3, 7, 8] or increasing the duration of the terminal buzz phase [9–11]. If such compensatory measures remain insufficient and there is no spectral and spatiotemporal release from masking noise, bats can also attempt to complete the task multiple times and spend more time before making decisions [7, 12]. Ultimately, however, when a primary sensory modality is compromised, a simple and tractable approach is to engage other concurrent sensory systems to increase the sensory redundancy of their surroundings [13, 14].

Ancestral bats are thought to have been small, visual predators that first evolved the ability to echolocate around 50–65 million years ago allowing access to the untapped niche of nocturnal, flying insects [11, 15]. The evolution of echolocation is still debated [16, 17], but over evolutionary time, echolocation became the dominant sensory modality to navigate and forage in darkness. Given the high metabolic cost for growth and repair of mammalian retinal tissue [18], the functionality of vision in small insectivorous bats may then be expected to be reduced or even lost, if their vision is unused [19]. Such reductions have been documented in other animal groups (i.e. some rodents, hagfish and cavefish), where vision has degenerated as an adaptation to perpetual darkness [20, 21]. This notion is supported by the evolutionary loss of echolocation in larger fruit-eating bats (Pteropodidae), which secondarily evolved large eyes with functional colour vision to exploit new diurnal foraging niches [22]. The apparent trade-off between investing in either echolocation or vision has likely resulted in large and non-insectivorous extant bats typically being more dependent on vision, whereas small insectivorous bats mostly rely on echolocation [22, 23]. Despite this, small extant insectivorous bats have maintained complex eye anatomy and display strong selection for the expression of photo-active opsin genes, suggesting functional vision [24, 25]. In support of this, both insectivorous and especially frugivorous bat species, have been found to multimodally supplement echolocation with vision in object recognition [26], obstacle avoidance [27–29], prey selection [30] and large-scale navigation [31].

Most insectivorous bat species are mainly active during the night, and otherwise spend most of their time in dark roosting sites. Despite their modest absolute eye size, small bat species are found to have many of the ocular adaptations to low-light vision typically found in nocturnal animals [32, 33]. These include scotopic vision mediated almost entirely through rod-cells [34] resulting in high sensitivity to brightness contrast in low-light conditions [35–37], but at the expense of visual acuity [23]. Accordingly, it has been suggested that echolocation is most useful for detecting small insects during foraging, whereas vision might be employed at longer ranges to augment long-range navigation [38]. However, Daubenton’s bats (*Myotis daubentonii*, Kuhl 1819) have high visual sensitivity and functional navigational vision at dusk [39] and a specialised retina thought to be advantageous when trawling insects close to the water surface [40]. This suggests that even small insectivorous bats, who rely heavily on echolocation, may still use vision for target range estimation when light cues are available.

Prompted by this possibility, we here investigated if small insectivorous bats dispense with or supplement echolocation with vision when solving an acoustically challenging task of target interception. We did this by training Daubenton’s bats to solve a landing task under several combinations of light and masking noise, thereby changing both the available visual information and the difficulty of acoustic problem-solving by echolocation. To make echolocation difficult, we exposed bats to masking noise high enough to render broadband echo-to-noise-ratios around zero dB or lower [6]. If bats solve the landing task by exploiting visual cues, we hypothesised that the bats (1) would solve the task faster and (2) would call less frequently (or cease echolocation entirely) in light conditions. However, if bats only rely on vision as the echolocation task becomes increasingly difficult in masking noise, we hypothesised that the bats (3) would rely less on adapting movement patterns, decreasing call intervals, prolonging buzz durations, or exhibiting a Lombard response, when visual information could replace the echoic information that is masked by noise.

## Methods

### Animal husbandry and training

**Fig. 1:**
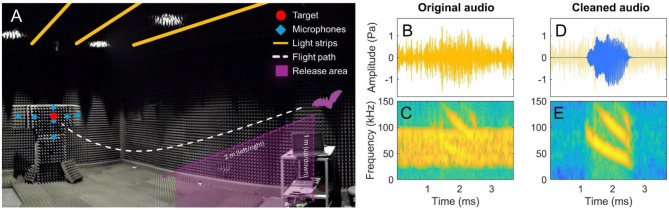
The experimental setting of the anechoic room. **(A)** Flight room with marked landing sphere (target, red circle) and surrounding microphone array (blue diamonds), randomised take-off position of bat from trainer within the release area (purple plane) and strips of LED lights (yellow lines). Photo: Ilias Foskolos. **(B,C)** Waveform and spectrogram of original audio with treatment noise overlapping a bat call. **(D,E)** Waveform and spectrogram of the same audio cleaned from treatment noise. Spectrogram settings: window length = 51, overlap = 50 samples, nfft = 500 points, sample rate = 400 kHz, dynamic range = 112 dB

For this study, we used five adult, male Daubenton’s bats (body mass 8.13 ± 0.72 g), which is a small insectivorous trawling bat found commonly in Northern Europe [41]. The bats were wild-caught during spring (2021) using mist-nets near Hobro, Denmark, and transferred to the animal care facilities of Aarhus University (permits: MST-850-00064 for capture of bats granted by the Danish Nature Agency (Skov- og Naturstyrelsen) and 2016-15-0201-00989 for animal experimentation granted by the national Animal Experiments Inspectorate (Dyreforsøgstilsynet) and the AICUC ethics committee at Aarhus University). The bats were kept at 18 ˚C and 60–80% humidity in a cloth-covered flight room (3 m x 1 m x 3 m) with a reversed 12 h:12 h day/night schedule. Bats had access to water (ad lib) and were fed mealworms either in bowls or a shallow trawling pool during rest days and as food rewards during data collection. Bats also received a high-calorie vitamin paste supplement (Nutri-cal, Tomlyn, Fort Worth, TX, USA) every week or as needed. Over a period of three weeks, we trained the bats to solve a navigational task consisting of taking off from the trainer’s hand, flying approximately four meters and landing on a spherical hydrophone acting as a landing target (Fig. 1A). Bats were trained using operant conditioning [42] and a “bridging” stimulus (soft tongue click by the trainer) followed by a food reward were given for correct behaviour (i.e. landing on the target). When training concluded, all bats consistently landed on the target within 30 s in all treatments. With the completion of experiments in late August (2021), all bats were released at the capture site after gradually being reintroduced to a natural diurnal cycle.

### Experimental design

During data collection, an experimenter and an animal trainer collected 30 landing trials/bat/day with interspersed rest days. The experimenter would trigger the onset of the recording, while the trainer, blinded to the noise condition, positioned the bat for take-off. The trainer pseudo-randomised the take-off position by moving left/right (approx. 2 m) and by moving the release position up/down (approx. 1 m) (Fig. 1A). When the bats took off voluntarily, the trainer pressed a handheld controller to timestamp the take-off. A subsequent “bridging” stimulus signalled the successful landing on the target and prompted the experimenter to stop the recording. The trainer then rewarded the bat and returned it to the starting position. To reward correct behaviour in the dark, the trainer watched a live-feed from an infra-red camera (TV-IP310PI, TRENDnet, Torrance, USA) mounted on the ceiling above the target.

Training and data collection took place in an anechoic room (Fig. 1A) lined with 12 cm deep pyramidal acoustic foam (30 dB echo reduction re. hard wall at frequencies 10–100 kHz). The spherical landing target was covered in light-grey fabric and was located at the centre-back of the room (120 cm above the ground). The target was protruding through a vertical plate covered in acoustic foam that carried a star-shaped array of six ultrasonic, Knowles microphones (FG-3329, 2.6 mm diameter, Itasca, IL, USA) mounted behind the target (Fig. 1A). Another microphone located just below the target recorded close to the acoustic axis of the bat calls. All microphone signals were amplified by custom-built filtering and gain box (1 pole, 1 kHz high-pass filter, 4 pole, 100 kHz anti-aliasing filter, 30 dB gain; Aarhus University Electronics workshop) before digitisation in an A/D-converter (USB-6356, 400 kHz, 16-bit, National Instruments, Houston, TX, USA). This seven microphone array facilitated 3D acoustic localisation with accurate localisations within distances up to 6–10 m from the array [6]. Verification of localisation was made using playback of ultrasonic sweeps from a speaker (Vifa, part #60,108, Avisoft Bioacoustics, Glienicke/Nordbahn, Germany) at known coordinates. In all cases, discrepancies between known and localised ranges resulted in transmission loss errors of less than 2 dB [6].

### Treatments

The target hydrophone (SF-26, Piezo Hannas, Wuhan, CN; 72 mm diameter, target strength (TS) − 15 dB at 0.1 m) was used as an omnidirectional ultrasonic transmitter of masking noise spanning the frequency range of the fundamental frequency of the bats’ echolocation calls (30–90 kHz) [6], thus securing the lack of spatiotemporal and spectral release from masking. The noise signal was amplified by a battery-powered amplifier (Marchand BE01 Piezo Transducer Amplifier, Rochester, NY, USA). After correcting for the transfer function of the hydrophone in air, the emitted spectrum was flat (± 2 dB) from 30 to 90 kHz *sensu* Pedersen et al. (2022) [43]. To facilitate the removal of noise during post-processing (see below), the noise was constructed circularly by repeating identical 1 s snippets of white noise. Additionally, the noise was generated at the same sampling rate (400 kHz) and on the exact clock timing as the sound recordings were recorded. The noise was constructed by inverse Fourier transformation of random samples in the relevant frequency range, which ensures that no glitches arose between repeating noise snippets. Noise treatments consisted of a silent control condition (10 dB re. 20µPa RMS at 0.1 m) and a noise condition (102 dB re. 20µPa RMS at 0.1 m). Noise levels (NL) were calibrated using a 1/8-inch microphone (46DP, GRAS Sound & Vibration, Holte, DK; sensitivity 156 dB re. 20 µPa V^− 1^).

To facilitate light treatments, four metal bars were mounted on the ceiling with warm white (V-TAC SMD 5050, SKU 2431, DC 24 V) and red (V-TAC VT-3528-60, DC 12 V) LED strips, giving a visually uniform lighting (Fig. 1A). A programmable power supply (RS PRO-3005P, RS, London, UK) placed outside the anechoic room powered the strips. Light treatments were calibrated using a handheld digital luxmeter (HT309, 0.01-400k lux, ± 3% accuracy, HT Instruments, Faenza, IT) held at the target while adjusting the voltage input of the light power supply. We used four light conditions consisting of “no light” (dark control, <<0.01 lx), “dim white light” (< 1 lx), “bright white light” (10 lx) and “red light” (10 lx). The red light condition was included to test the hypothesis that bats cannot see red light, which commonly results in use of red light with bats both in captivity and in the wild. We checked the spectra of the LEDs using a spectral light meter (Gigahertz-Optik, MSC15, Munich, DE), which showed expected values (Figure S1). All other light sources than those facilitating the experimental light conditions were minimised. The bats were tested on all light conditions interchangeably on a randomised schedule. Inside the anechoic chamber, a custom program implemented in LabVIEW (v.2015f, National Instruments, TX, USA) was used by the experimenter to change noise and light conditions during data collection.

### Data analysis

Call detection was done with a custom configuration of PAMGuard (BETA-version 2.01.05 with SMRU modules) [44]. Time-of-arrival differences for each recorded call on different microphones were estimated using cross-correlation between channels. By using a simplex minimisation algorithm in MATLAB (R2020a, Mathworks Inc., Natick, MA), these time-of-arrival differences allowed localisation and range estimation of bats in 3D space for each call emission [45]. All localisations were checked visually. Ranges of incorrect localisations were interpolated from nearby locations. When bats took multiple attempts to land, only calls from the final approach were included in further analysis. As we did not have an exact measure of the time of landing, we defined “time to land” (TTL) as the difference between the animal trainer’s timestamp of the take-off and the time of the final call in the buzz. Trainer reaction times are assumed to vary naturally by 180–200 ms [46], so some error is expected. Non-normality of TTLs with multiple peaks (e.g. 1.2 s, 4.5 s and 8 s) reflected how bats either landed straight from the hand or circled the flight room and passed the target one or more times before landing (Figure S3). Call intervals (CI) were calculated as the time difference in a call’s onset and the following call’s onset. Buzz onset range was defined at the call before the first two subsequent calls with CIs < 15 ms [9]. Buzz duration was then calculated per trial as the time difference between the first and last buzz call.

The estimated range (R) from bat to target allowed calculation of source levels (SL) using the received levels (RL) of the calls. We used a sound attenuation model following 20 log_10_ (R/R_ref_) [6], where reference distance R_ref_ = 0.1 m and we estimated atmospheric absorption assuming 20˚C and 70% RH at R_ref_ [47]. RLs were quantified as RMS amplitude within the time window defined by the − 10 dB points of the signal envelope peak and call duration of the same time window allowed estimation of RLs in energy flux density units (i.e. dB re. 20µPa^2^ s).

To improve call detection when masking noise resulted in poor SNRs of the recorded calls, we performed call detections in noise trials after removing the noise (Fig. 1B-E). First, audio files with noise exposure were shortened to a length *n* (integer) seconds, therefore spanning exactly *n* repeated noise snippets. This audio waveform was then split into *n* 1-second waveforms, each containing exactly one cycle of treatment noise overlaying natural noise and echolocation calls. The median of the sample values across all 1-second chunks was used as a template of the treatment noise. By repeating the median noise snippet *n* times to make a full-length noise template, we then subtracted it from the original, noisy signal waveform. This resulted in an almost noise-free waveform (Fig. 1D-E) rendering call detection and RL estimation possible. For all trials, SLs were then estimated by adding the attenuation and absorption to the calculated RLs. We found an estimated error of < 1 dB over the range of SNRs relevant to the actual SNRs in our noise trials (Figure S2).

The Lombard response magnitude was found as the ratio between the change in emitted SLs during noise and the change in received NL during noise (ΔSL_emitted_ / ΔNL_received_). We estimated the bats’ received NL at the mean range of all calls emitted in noise by using measured sound attenuation of 20 log_10_(R) and absorption. For control trials without noise exposure, we assumed the bats’ received NL to equal the noise-floor of their auditory system (i.e. 20 dB re. 20µPa RMS), based on the echo detection threshold of vespertilionid bats [48]. We estimated the error of the Lombard response magnitude by bootstrapping [49] the calculation per light conditions (1000 reps × 100 pseudo samples) to calculate 95% quantiles. Additionally, we estimated the magnitude of SL compensation at different stages in the approach by binning range data logarithmically against SL and finding the magnitude of SL compensation to range (*magnitude × log*_*10*_*(range)*) per bin using linear regression (*magnitude × log*_*10*_*(range) + intercept*).

### Statistical analysis

All statistical analyses were done in MATLAB. From the experimental design phase, we intended on analysing all data using Linear Mixed Effects Models (LMMs) to account for the individual and temporal autocorrelation. Since a high number of replicates resulted inflated p-values for the call data [50], we here decided to determine biological significance using comparison of means and quantiles except for modelling of the time to land.

To test our first hypothesis, we investigated the relationship between the time the bats spend completing the task in different noise and light conditions. We used time to land (TTL) as a response variable, explained by noise, light condition and bat ID as categorical fixed effect variables and date was included as a categorical random effect. We used only trials where the bats landed in the first pass defined as TTLs below 3 s (1149 out of 1365 trials), which was determined using the minimum TTL between the two first bimodal peaks of the TTL histogram (Figure S3). We examined the residuals of the models, and found that the model met assumptions of normality, homoscedasticity, and independence (R^2^ = 0.17).

To test our second hypothesis, we examined how bats used decreased call intervals and increased buzz durations in different light and noise conditions. As a result of the coupling between call production and wingbeat cycle in bats [51, 52], we found bimodal CI distributions. To identify individual peaks in CI data, we performed *k-*means clustering on all non-buzz calls using the function *kmeans()* with 4 clusters. To quantify the range where bats switched between producing calls in different CI clusters, we used binomial regression (link = logit) on binary data of the two most prominent data clusters. This was done on individual bat and light condition subsets to evaluate the effects of these variables. From the binomial regression, the inflection point (p50) and 95% confidence intervals allowed us to estimate the range at which bats started increasing the number of calls per wingbeat.

## Results

During autumn 2020, we collected acoustic data from five Daubenton’s bats approaching a target over a period of ten days. After filtering the call data and deleting any faulty call detections in the noise, the final dataset consisted of 1365 trials with 62,820 calls. Silent controls (*n* = 787) and noise trials (*n* = 578) both contained an average of 46 calls (Figure S4A) and had similar range distributions (Figure S4B).

### Time to land

**Fig. 2:**
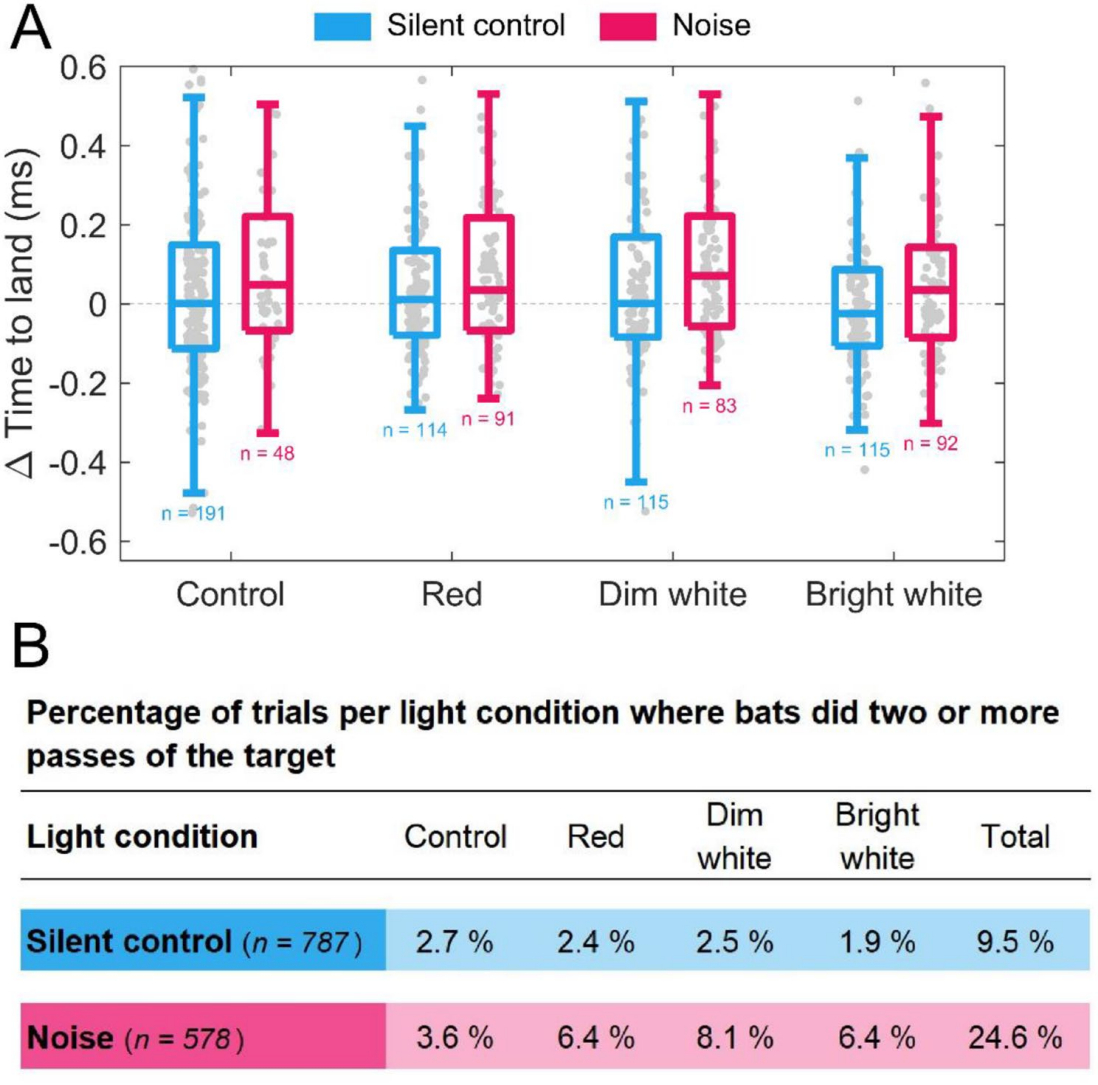
Time to land (TTL) under different light and noise treatments, where ΔTTL is the change in TTL with respect to the mean TTL per bat in silent controls. **(A)** Boxplots of TTL when bats landed on the first pass of the target. Below the boxplots is the number of datapoints (n) pooled for all bats in each treatment. **(B)** Table showing the proportion of trials in which bats used multiple passes before landing on the target per light treatment, given in percentages of total number of silent controls or noise trials respectively

First, we tested the hypothesis that the bats would solve the task in less time if they have access to visual cues and can gather visual information to solve the task. However, we found no evidence of bats using visual cues to decrease their time to land (TTL). The bats solved the task in 1.34 s [0.98, 2.30] in noise compared to in 1.31 s [0.93, 2.24] during silent controls, where most variation was between individuals (Table S1). Furthermore, the light treatments had no effect on TTL (red: 34.3 ms ± SE 27.7 / *p* = 0.22, dim white: -4.8 ms ± SE 27.9 / *p* = 0.86, bright white: 11.2 ms ± SE 27.4 / *p* = 0.68, Fig. 2A) and neither did noise conditions (21.4 ms ± SE 17.0 / *p* = 0.21, Fig. 2A + Table S1). However, four of the five bats (Table S2) were more likely to perform multiple rounds in the flight room before landing during noise exposure (24.6% in noise trials vs. 9.5% in silent controls, Fig. 2B).

### Call interval and buzz duration

**Fig. 3:**
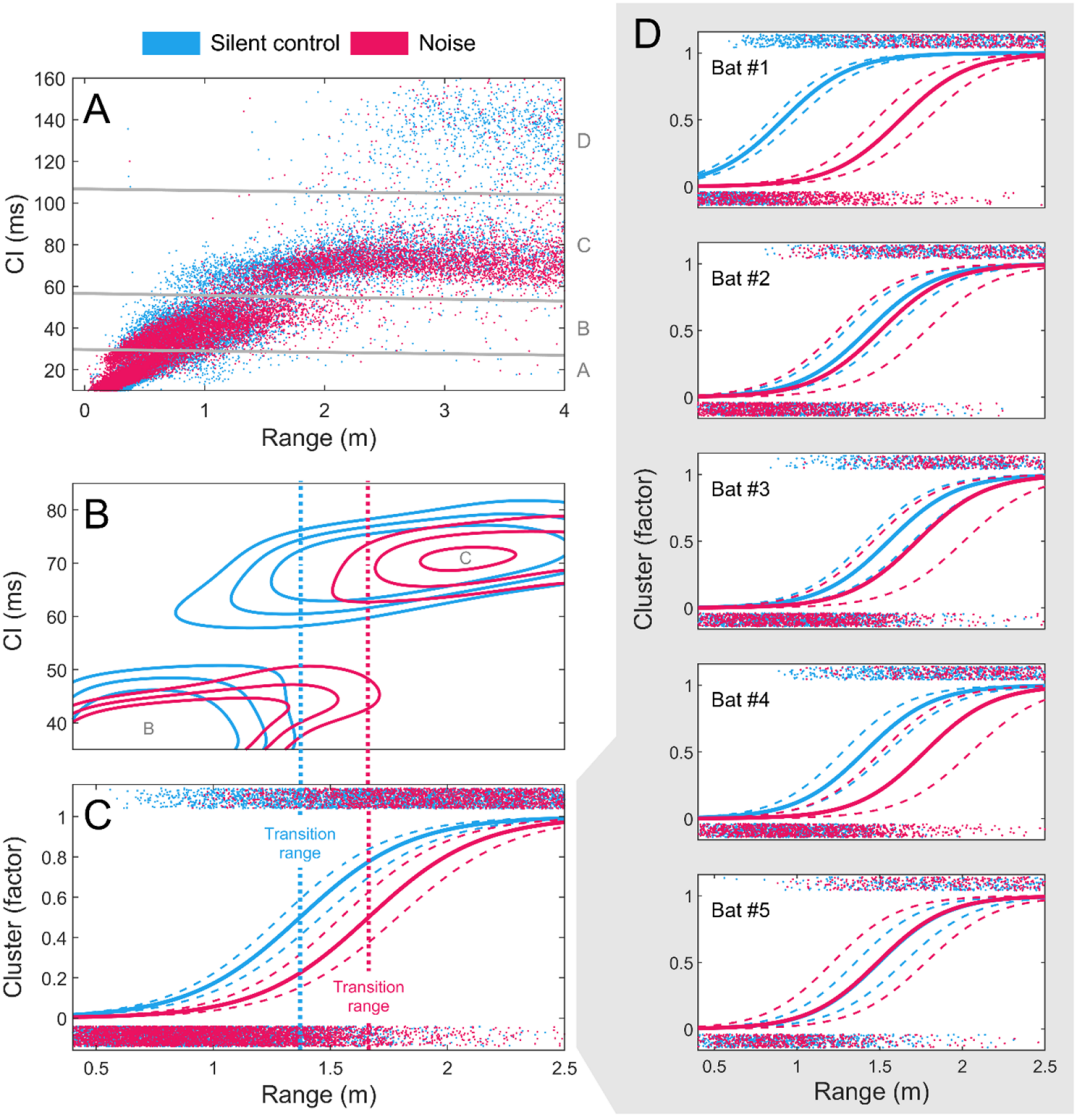
Call intervals under different light and noise treatments for all bats grouped (panels A-C) and for individual bats (panel D). **(A)** Scatter plots of call intervals for both noise combinations (pooled over bat IDs and light conditions), showing the four clusters of call intervals. Grey lines separate data clusters found by k-means (k = 4) clustering of all approach calls, and grey letters (right y-axis) name clusters. **(B)** Contour plots of cluster B (bottom left) and cluster C (top right) in call intervals against range. **(C)** Binomial logistic regression on clusters B and C against range for all bats (solid lines) with 95% confidence intervals (broken lines). Point clouds in top and bottom show raw cluster/range data. Dotted lines indicate the p50 value (range of switching between clusters), and corresponding range on panel B. **(D)** Binomial logistic regression for individual bats (refer to description of panel C)

Next, we tested the hypothesis that the bats would dispense with echolocation entirely when visual information was available. However, we found that the bats continued to echolocate throughout the experiment regardless of light condition, and we therefore investigated whether the bats instead would call less in different light conditions. All call intervals across noise and light treatments showed a multimodal distribution with four consistent data peaks corresponding to a buzz call peak (CI: 7.1 ms [5.2, 13.9]) and seemingly three approach/search call peaks. K-means clustering identified a total of four approach call clusters with mean CIs of 21 ms [13.2, 28.7], 40.3 ms [30.6, 51.0], 71 ms [57.4, 85.1] and 145.9 ms [107.7, 199.6] (hereafter named: cluster A, B, C and D, Fig. 3A). Assuming a wingbeat frequency of 12–16 wingbeats/second for Daubenton’s bats [53], these clusters correspond reasonably well with wingbeat couplings of 3.5, 2, 1 and 0.5 calls/wingbeat respectively. Logistic modelling of cluster B and cluster C as binary categories showed that at least two of the five bats (Fig. 3D) would start calling faster earlier in the approach in noise (1.66 m, [1.55, 1.79]_95%_) compared to in silent controls (1.37 m, [1.29, 1.46]_95%_) (Fig. 3B-C). Despite a high inter-individual variation, this pattern was similar across all light conditions (Figure S5). Therefore, we found no evidence of bats calling less when visual cues were available.

Since bats call more often in noisy conditions, we wanted to test whether the bats would also change the duration of their buzzes. In four of the five bats, mean buzz durations were similar in noise (154.8 ms [129.8, 237.6]) and in silent controls (181.2 ms [82.2, 225.2]) and consistent across light conditions (Table S4). However, in individual #4, buzz duration decreased from 173.7 ms [129.8, 221.8] to 104.3 ms [53.7, 145.9] in noise, which was likely explained by a concomitant delay of the buzz onset (Table S5).

### Source level

**Fig. 4:**
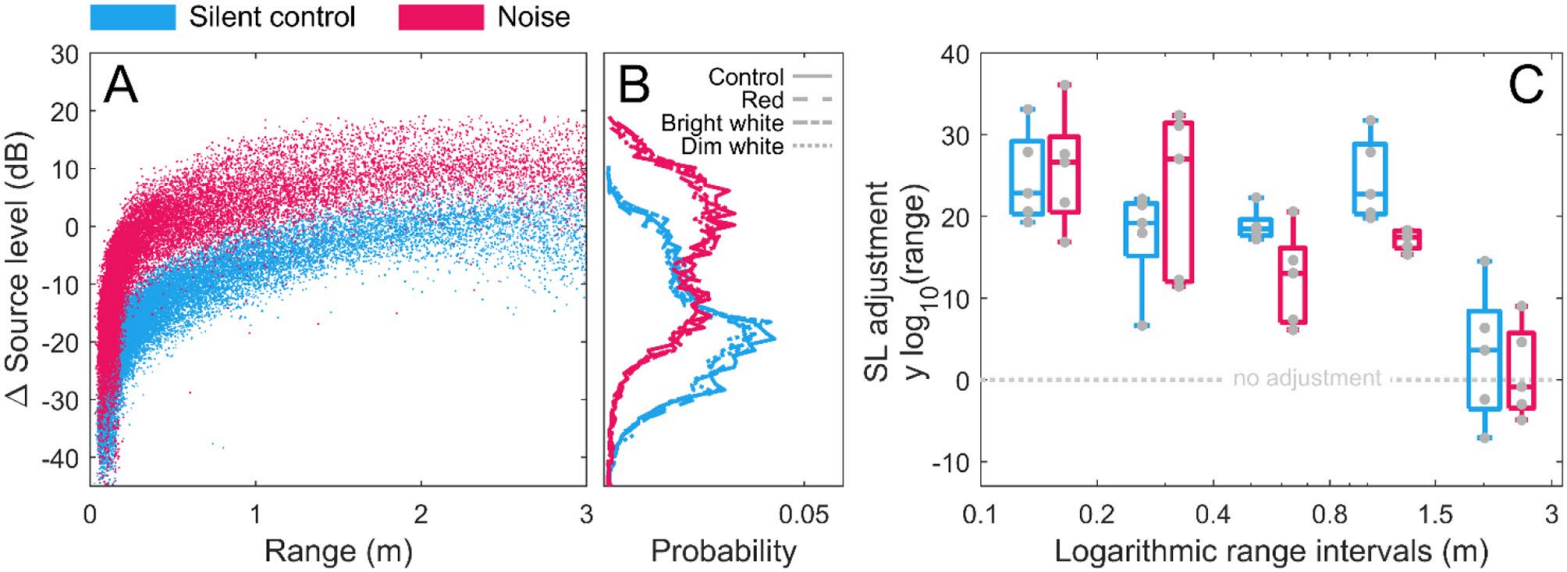
Call source levels under different light and noise treatments. **(A)** Scatterplot of the change in source levels by noise condition, where ΔSL is the change in SL with respect to the mean SL (at ranges 2–4 m) per bat in silent controls. **(B)** Probability histograms of call source levels for all light and noise combinations, showing the Lombard response shift of SL in noise treatments (also shown in A). **(C)** Magnitude of the logarithmic SL compensation with range, shown as the slope of the linear regression of SL versus log_10_(range). Boxplots are based on individual means (*n* = 5 bats, grey points) and are spaced in logarithmic range bins

Finally, we tested the hypothesis that the bats reduce their Lombard response when they can exploit visual cues. We found an increase in SLs when bats were subjected to noise (64.6 dB re. 20µPa^2^s at 0.1 m [42.0, 81.6]) compared to silent controls (52.0 dB re. 20µPa^2^s at 0.1 m [34.3, 71.9]) corresponding to a Lombard response of 0.18 dB/dB_noise_ [0.14, 0.22] in dark controls (Fig. 4A). The Lombard response remained unaffected by range (Fig. 4A) and light treatment (red: 0.18 dB/dB [0.14, 0.22]), dim white: 0.18 dB/dB [0.13, 0.21], bright white: 0.18 dB/dB [0.14, 0.22]), and varied only slightly between individuals (Table S6). Since the received noise level increases during the flight towards the target, we then wanted to test whether the SL compensation changed with respect to the range at call emission in light. The magnitude of SL compensation varied with range and reached a maximal SL adjustment of around 25 log_10_(range) just before landing regardless of noise conditions (Fig. 4C). While all bats started decreasing SLs at 1.5 m target range regardless of noise condition, the bats subjected to noise adjusted SL more heavily later in the approach (0.2–0.4 m) compared to in silent controls (0.8–1.5 m) (Fig. 4C). Light condition did not affect this pattern (Fig. 4B + S7).

## Discussion

Echolocating bats navigate dark and complex environments while flying at high speeds and must therefore continually gather enough information to make rapid decisions based on small sensory volumes [54]. Here we ask if this active sensory system can be replaced by vision in insectivorous bats when visual information is available and echolocation is difficult? To answer this question, we exposed bats to masking noise and different light treatments while they were trained to solve a landing task. Since the noise treatments produced near-zero or even negative broad-band ENRs [6], their sonar-based navigation was substantially impaired. Therefore, this setup should represent a scenario where the ability to echolocate was pushed to its limits, prompting a strong incentive to gather information via other sensory modalities. Under these conditions, we hypothesised that bats could exploit visual cues in light conditions, allowing them to partially or entirely dispense with echolocation. We reject this hypothesis by finding that the bats continued to echolocate stereotypically throughout all experimental treatments and therefore did not dispense with echolocation in any light and noise condition.

### Bats do not solve the landing task faster when light is available

Since small echolocating bats have maintained sizeable eyes and high-resolution vision, we next tested the hypothesis that the bats could therefore solve the task faster in light conditions than in darkness. We found no evidence that the bats adjusted flight speed nor solved the task faster when flying in light conditions (Fig. 2). This suggests that the bats did not benefit from additional visual input to guide their target approach behaviour even when noise conditions compromised their echolocation performance. The bats could to some extend have relied on spatial memory or used the target as a beacon as an additional source of information to guide their landings irrespective of noise and light conditions. However, we find this explanation unlikely since we both randomised release positions and performed two previous control experiments. In the first experiment, trials were conducted with a noise source uncoupled from the landing target to remove potential homing cues. In the second experiment, the recording array and target were repeatedly moved 1.5 m left/right to circumvent the use of spatial memory. Throughout both experiments, the bats continued to land and echolocate successfully, suggesting that spatial memory or homing alone cannot explain how the bats solved the task [6]. In noisy conditions, the bats were more likely to approach the target multiple times irrespective of light conditions supporting that the task was indeed solved with echolocation as the primary modality. The multiple rounds of flight in the room before landing on the target is in contrast to blindfolded echolocating porpoises that decrease swim speeds when only echolocation cues are available [55]. However, in comparison to toothed whales, bats are constrained by a slower speed of sound in air, a strict coupling of their call emissions to wingbeats to produce calls efficiently, and a need to stay airborne via lift generated from forward motion, which may explain why bats approach the target multiple times instead of decreasing their closing speed towards the target. These different strategies across bats and toothed whales are nevertheless functionally similar since it allows both groups of echolocating animals to gather more sensory information per distance to solve a task in complex environments.

### Bats compensate with decreased call intervals in noise, but do not increase buzz duration

Even though the bats solved the acoustically challenging task at similar speeds in light and darkness, the bats might have relied less on echolocation by decreasing their sensory sampling in light conditions. To our surprise, some of the bats instead increased their sensory sampling by decreasing call intervals in noise even under light conditions (Fig. 3) where they could have relied on visual information instead. This suggests that the bats did not improve their sensory redundancy by integrating visual information to solve their task at hand. Instead, some of the bats adjusted their echolocation behaviour by decreasing their call intervals when exposed to noise akin to what has been found in previous studies from laboratory experiments on wild-caught Daubenton’s bats exposed to noise while pursuing prey [7, 56]. However, these two studies exposed the bats to noise that did not spectrally overlap with the echolocation calls, suggesting that increased sensory sampling might have been promoted by noise-induced distraction from the task rather than masking. Increased call rates have also been found as a clutter-rejection response in several bat species [57–59], indicating that this mechanism is generally used in acoustically complex environments. Echolocating toothed whales employ similar acoustic adjustments to complex tasks [60, 61] showing functional convergence in the bio-sonar systems of these two mammalian clades.

When solving challenging tasks such as during prey tracking, different species of bats and toothed whales have also been shown to increase their available sensory information by prolonging the duration of their buzzes [11, 62, 63]. Since the bats increased their sensory sampling in response to noise, but not light conditions, we next sought to investigate whether the bats would similarly increase their sensory information by prolonging their buzz in response to masking noise in darkness, but not in lighted conditions. We found no increases in buzz duration nor buzz onset range in response to masking noise and light conditions did not change this pattern. This suggests that prolongation of buzz durations in bats might be a context-dependent adjustment to tracking of prey [11] rather than a reflection of the task difficulty [63].

### Bats exhibit a consistent range adjustment in call levels and an inflexible Lombard reflex

When approaching a target, bats both in the wild and in captivity reduce their call source levels to receive echoes within a manageable dynamic range of the auditory system [54]. We found that the bats decreased SLs when approaching the target in both silent controls and noisy conditions (Fig. 4). The steepest decreases in SLs were found later in the approach in noise compared to during silent controls, which is like the pattern found in Pratt’s roundleaf bats (*Hipposideros pratti*) where it is suggested that echo feedback mediates SL adjustments to range [64]. Since the bats also decreased SLs with range in noisy conditions, we next hypothesised that in a lit environment with poor ENRs, the bats would dispense with range-dependent adjustments in SL when visual information could replace echoic information. We tested this by investigating whether the SLs adjustments to range differed across light treatments. We found no change in the SL compensation over all ranges between light and dark conditions and across individual bats, showing that the bats adjusted their call SLs to range no matter if light cues where available (Fig. 4C). This supports the interpretation that the general biosonar adjustments employed by these small insectivorous bats are hard-wired and that echolocation has a strong sensory priority at least when solving close-range navigational tasks. Similarly, we hypothesised that bats in noisy and lighted conditions would dispense with increasing call source levels in response to increasing noise (i.e. the Lombard response). However, we reject that hypothesis by finding that the bats maintained a consistent Lombard response of 0.18 dB/dB [0.14, 0.22] when subjected to noise across all light and dark conditions and varied only marginally between individual bats. This inflexibility and consistency in the bats’ overall source level adjustments even in situations where vision is available, adds to the growing evidence that the Lombard response is an ancestral vertebrate trait [65, 66] and a subcortical reflex outside cognitive control [67–69].

In contrast to the overall call source level adjustments and Lombard responses that were highly consistent across individuals, we find considerable inter-individual variability in other acoustic behaviours in response to noise. Out of five individuals, two individuals decreased their call intervals earlier in the approach (Fig. 3D), one individual reduced its buzz durations (Figure S6), and four individuals attempted the task multiple times more frequently during noise exposure (Table S2). These adjustments may therefore in contrast to the Lombard response be the result of higher-level cortical processes that are learned or adapted perhaps through vocal learning [70], explaining the high inter-individual differences.

### Conclusion: bats show stereotyped and hard-wired echolocation behaviour

In this study we sought to test whether echolocating bats faced with noise would rely on multimodal sensing by switching fully or partially to visual navigation and dispensing with echolocation. Overall, we found no evidence that our studied Daubenton’s bats augment echolocation cues with visual information. The individual bats used the same echolocation strategies and behaviour in masking noise, even when visual cues could replace or complement compromised echo information. However, as our results do not strictly allow us to exclude the possibility that bats use visual cues to complement echolocation, we suggest two possible explanations for the observed responses to light and noise: (1) bats *do not* utilise vision for this task, so echolocation behaviour remains unchanged even when faced with potentially distracting light conditions, or (2) bats *do* utilise vision to solve the task, but continue to employ stereotyped echolocation. Our finding that bats in light and noise employ an unchanged and stereotyped echolocation behaviour have implications for the interpretation of acoustic data in laboratory experiments with bats. Laboratory experiments are often performed in darkness to exclude the use of vision in echolocation studies. Species of the genus *Myotis*, like Daubenton’s bats, are generally considered to be light intolerant [71] and in our experiment they were exposed to relatively intense light conditions. Despite this, the bats maintained unchanged acoustic behaviour irrespective of light conditions, suggesting that these bats are either unaffected, desensitised or that potential effects of light exposure might not be evident in acoustic data.

We show that echolocation in a small insectivorous bat species is a sensory system with a wide range of compensation strategies to masking noise. Some differ between individuals such as adjustments to call intervals and movement patterns, and some strategies such as source level adjustments and Lombard responses are highly consistent across individuals. Nonetheless, all these adjustments when navigating noisy spaces remain stereotyped irrespective of light conditions, suggesting a hard-wired echolocation behaviour regardless of the availability of visual cues. Thus, in contrast to the multimodality employed by frugivorous bats in situations where they could rely on visual stimuli, acoustic compensation strategies in insectivorous bats remain stereotyped and reflex-like even in lighted conditions. Despite their high visual sensitivity, we find no evidence to support the idea that these bats do not rely on vision in small-scale navigational tasks. They may instead rely on vision for navigation, and multimodal sensory integration during more complex behavioural task such as prey tracking. We conclude that insectivorous bats likely do not use vision to solve short range echolocation tasks, but stress that our results do not alleviate the growing concerns about effects of light pollution on wild, insectivorous bats.

### Electronic supplementary material

Below is the link to the electronic supplementary material.

Supplementary Material 1

## Data Availability

The datasets used and/or analysed during the current study are available in supplemental materials or from the corresponding author on reasonable request.

## Abbreviations

TTL: Time to land
CI: call interval
R: call range
R_ref_: reference distance (0.1 m)
RMS: root-mean-square
RL: received level
SL: source level
TS: target strength
NL: noise level
SNR: signal-to-noise ratio
ENR: echo-to-noise ratio

## References

[1] Jones TK, Allen KM, Moss CF (2021). Communication with self, friends and foes in active-sensing animals. J Exp Biol.

[2] Griffin DR, Webster FA, Michael CR (1960). The echolocation of flying insects by bats. Anim Behav.

[3] Brumm H, Slabbekoorn H. (2005) Acoustic Communication in Noise. In: Advances in the Study of Behavior. Academic Press, pp 151–209.

[4] Lombard E (1911). Le Signe De l’élévation De La Voix. Ann Malad l’Oreille Larynx.

[5] Tressler J, Smotherman MS (2009). Context-dependent effects of noise on echolocation pulse characteristics in free-tailed bats. J Comp Physiol Neuroethol Sens Neural Behav Physiol.

[6] Foskolos I, Bjerre Pedersen M, Beedholm K, Uebel AS, Macaulay J, Stidsholt L, Brinkløv S, Madsen PT (2022). Echolocating Daubenton’s bats are resilient to broadband, ultrasonic masking noise during active target approaches. J Exp Biol.

[7] Allen LC, Hristov NI, Rubin JJ, Lightsey JT, Barber JR. (2021) Noise distracts foraging bats. Proceedings of the Royal Society B: Biological Sciences 288:20202689. 10.1098/rspb.2020.2689 PMC7893219 33563124

[8] Luo J, Goerlitz HR, Brumm H, Wiegrebe L (2015). Linking the sender to the receiver: vocal adjustments by bats to maintain signal detection in noise. Sci Rep.

[9] Geberl C, Brinkløv S, Wiegrebe L, Surlykke A (2015). Fast sensory–motor reactions in echolocating bats to sudden changes during the final buzz and prey intercept. Proc Natl Acad Sci.

[10] Moss CF, Bohn K, Gilkenson H, Surlykke A (2006). Active listening for spatial orientation in a Complex Auditory Scene. PLoS Biol.

[11] Ratcliffe JM, Elemans CPH, Jakobsen L, Surlykke A (2013). How the bat got its buzz. Biol Lett.

[12] Gomes DGE, Page RA, Geipel I, Taylor RC, Ryan MJ, Halfwerk W (2016). Bats perceptually weight prey cues across sensory systems when hunting in noise. Science.

[13] Schumacher S, Perera T, Thenert J, Emde G (2016). Cross-modal object recognition and dynamic weighting of sensory inputs in a fish. Proc Natl Acad Sci.

[14] Sheppard JP, Raposo D, Churchland AK (2013). Dynamic weighting of multisensory stimuli shapes decision-making in rats and humans. J Vis.

[15] Teeling EC, Springer MS, Madsen O, Bates P, O’Brien SJ, Murphy WJ (2005). A molecular phylogeny for bats illuminates Biogeography and the Fossil Record. Science.

[16] Hand SJ, Maugoust J, Beck RMD, Orliac MJ (2023). A 50-million-year-old, three-dimensionally preserved bat skull supports an early origin for modern echolocation. Curr Biol.

[17] Simmons NB, Seymour KL, Habersetzer J, Gunnell GF (2008). Primitive early eocene bat from Wyoming and the evolution of flight and echolocation. Nature.

[18] Ng SK, Wood JPM, Chidlow G, Han G, Kittipassorn T, Peet DJ, Casson RJ (2015). Cancer-like metabolism of the mammalian retina. Clin Exp Ophthalmol.

[19] Niven JE, Laughlin SB (2008). Energy limitation as a selective pressure on the evolution of sensory systems. J Exp Biol.

[20] Moran D, Softley R, Warrant EJ (2015). The energetic cost of vision and the evolution of eyeless Mexican cavefish. Sci Adv.

[21] Sanyal S, Jansen HG, de Grip WJ, Nevo E, de Jong WW (1990). The eye of the blind mole rat, Spalax ehrenbergi. Rudiment with hidden function?. Investig Ophthalmol Vis Sci.

[22] Thiagavel J, Cechetto C, Santana SE, Jakobsen L, Warrant EJ, Ratcliffe JM (2018). Auditory opportunity and visual constraint enabled the evolution of echolocation in bats. Nat Commun.

[23] Eklöf JS, Pētersons G, Rydell J. Visual acuity and eye size in five European bat species in relation to foraging and migration strategies. Environmental and Experimental Biology; 2014. pp. 1–6.

[24] Shen Y-Y, Liu J, Irwin DM, Zhang Y-P (2010). Parallel and convergent evolution of the Dim-Light Vision Gene RH1 in bats (Order: Chiroptera). PLoS ONE.

[25] Zhao H, Xu D, Zhou Y, Flanders J, Zhang S (2009). Evolution of opsin genes reveals a functional role of vision in the echolocating little brown bat (Myotis lucifugus). Biochem Syst Ecol.

[26] Danilovich S, Yovel Y (2019). Integrating vision and echolocation for navigation and perception in bats. Sci Adv.

[27] Jones TK, Moss CF (2021). Visual cues enhance obstacle avoidance in echolocating bats. J Exp Biol.

[28] Kugler K, Luksch H, Peremans H, Vanderelst D, Wiegrebe L, Firzlaff U (2019). Optic and echo-acoustic flow interact in bats. J Exp Biol.

[29] Orbach DN, Fenton B (2010). Vision impairs the abilities of bats to avoid colliding with stationary obstacles. PLoS ONE.

[30] Leavell B, Rubin J, McClure C, Miner K, Branham M, Barber J (2018). Fireflies thwart bat attack with multisensory warnings. Sci Adv.

[31] Tsoar A, Nathan R, Bartan Y, Vyssotski A, Dell’Omo G, Ulanovsky N. (2011) Large-scale navigational map in a mammal. Proceedings of the National Academy of Sciences 108:E718–E724. 10.1073/pnas.1107365108 PMC3174628 21844350

[32] Jeon C-J, Strettoi E, Masland RH (1998). The major cell populations of the Mouse Retina. J Neurosci.

[33] Orlowski J, Harmening W, Wagner H (2012). Night vision in barn owls: visual acuity and contrast sensitivity under dark adaptation. J Vis.

[34] Suthers RA, Wallis NE (1970). Optics of the eyes of echolocating bats. Vision Res.

[35] Hope GM, Bhatnagar KP (1979). Effect of light adaptation on electrical responses of the retinas of four species of bats. Experientia.

[36] Masterson FA, Ellins SR (1974). The role of Vision in the orientation of the Echolocating Bat, Myotis lucifugus. Behaviour.

[37] Müller B, Glösmann M, Peichl L, Knop GC, Hagemann C, Ammermüller J (2009). Bat eyes have Ultraviolet-sensitive cone photoreceptors. PLoS ONE.

[38] Boonman A, Bar-On Y, Yovel Y (2013). It’s not black or white—on the range of vision and echolocation in echolocating bats. Front Physiol.

[39] Céchetto C, Jakobsen L, Warrant EJ (2023). Visual detection threshold in the echolocating Daubenton’s bat (Myotis daubentonii). J Exp Biol.

[40] Cechetto C, de Busserolles F, Jakobsen L, Warrant EJ. (2020) Retinal Ganglion Cell Topography and Spatial Resolving Power in Echolocating and Non-Echolocating Bats. BBE 1–11. 10.1159/000508863 32818939

[41] Encarnação JA, Becker NI, Hackländer K, Zachos FE (2020). Daubenton’s Bat Myotis daubentonii (Kuhl, 1817). Handbook of the mammals of Europe.

[42] Staddon JER, Cerutti DT (2003). Operant conditioning. Annu Rev Psychol.

[43] Pedersen MB, Uebel AS, Beedholm K, Foskolos I, Stidsholt L, Madsen PT (2022). Echolocating Daubenton’s bats call louder, but show no spectral jamming avoidance in response to bands of masking noise during a landing task. J Exp Biol.

[44] Gillespie D, Mellinger DK, Gordon J, McLaren D, Redmond P, McHugh R, Trinder P, Deng X, Thode A (2009). PAMGUARD: Semiautomated, open source software for real-time acoustic detection and localization of cetaceans. J Acoust Soc Am.

[45] Nelder JA, Mead R (1965). A simplex method for function minimization. Comput J.

[46] Thompson PD, Colebatch JG, Brown P, Rothwell JC, Day BL, Obeso JA, Marsden CD (1992). Voluntary stimulus-sensitive jerks and jumps mimicking myoclonus or pathological startle syndromes. Mov Disord.

[47] Bass HE, Sutherland LC, Zuckerwar AJ, Blackstock DT, Hester DM (1995). Atmospheric absorption of sound: further developments. J Acoust Soc Am.

[48] Stilz W-P, Schnitzler H-U (2012). Estimation of the acoustic range of bat echolocation for extended targets. J Acoust Soc Am.

[49] Efron B (1979). Bootstrap methods: another look at the Jackknife. Annals Stat.

[50] Lin M, Lucas HC, Shmueli G (2013). Research Commentary—too big to fail: large samples and the p-Value Problem. Inform Syst Res.

[51] Stidsholt L, Johnson M, Goerlitz HR, Madsen PT (2021). Wild bats briefly decouple sound production from wingbeats to increase sensory flow during prey captures. iScience.

[52] Suthers RA, Thomas SP, Suthers BJ (1972). Respiration, Wing-beat and Ultrasonic Pulse Emission in an Echo-locating Bat. J Exp Biol.

[53] Kalko EKV, Schnitzler H-U (1989). The echolocation and hunting behavior of Daubenton’s bat, Myotis daubentoni. Behav Ecol Sociobiol.

[54] Stidsholt L, Greif S, Goerlitz HR, Beedholm K, Macaulay J, Johnson M, Madsen PT (2021). Hunting bats adjust their echolocation to receive weak prey echoes for clutter reduction. Sci Adv.

[55] Verfuß UK, Miller LA, Pilz PKD, Schnitzler H-U (2009). Echolocation by two foraging harbour porpoises (Phocoena phocoena). J Exp Biol.

[56] Luo J, Siemers BM, Koselj K (2015). How anthropogenic noise affects foraging. Glob Change Biol.

[57] Beetz MJ, Kössl M, Hechavarría JC (2019). Adaptations in the call emission pattern of frugivorous bats when orienting under challenging conditions. J Comp Physiol A.

[58] Petrites AE, Eng OS, Mowlds DS, Simmons JA, DeLong CM (2009). Interpulse interval modulation by echolocating big brown bats (Eptesicus fuscus) in different densities of obstacle clutter. J Comp Physiol A.

[59] Wheeler AR, Fulton KA, Gaudette JE, Simmons RA, Matsuo I, Simmons JA. (2016) Echolocating Big Brown bats, Eptesicus fuscus, modulate pulse intervals to overcome Range Ambiguity in Cluttered surroundings. Front Behav Neurosci. 10.3389/fnbeh.2016.00125. 10.3389/fnbeh.2016.00125 PMC4916216 27445723

[60] Buckstaff KC (2004). Effects of Watercraft noise on the Acoustic Behavior of Bottlenose Dolphins, Tursiops Truncatus, in Sarasota Bay, Florida. Mar Mamm Sci.

[61] Ladegaard M, Madsen PT. (2019) Context-dependent biosonar adjustments during active target approaches in echolocating harbour porpoises. J Experimental Biology Jeb.206169. 10.1242/jeb.206169 31350302

[62] Fais A, Johnson M, Wilson M, Aguilar Soto N, Madsen PT (2016). Sperm whale predator-prey interactions involve chasing and buzzing, but no acoustic stunning. Sci Rep.

[63] Hulgard K, Ratcliffe JM (2016). Sonar sound groups and increased terminal buzz duration reflect task complexity in hunting bats. Sci Rep.

[64] Luo J, Lu M, Luo J, Moss CF (2023). Echo feedback mediates noise-induced vocal modifications in flying bats. J Comp Physiol A.

[65] Kunc HP, Morrison K, Schmidt R. (2022) A meta-analysis on the evolution of the Lombard effect reveals that amplitude adjustments are a widespread vertebrate mechanism. Proceedings of the National Academy of Sciences 119:e2117809119. 10.1073/pnas.2117809119 PMC9335264 35858414

[66] Luo J, Hage SR, Moss CF (2018). The Lombard Effect: from acoustics to neural mechanisms. Trends Neurosci.

[67] Nonaka S, Takahashi R, Enomoto K, Katada A, Unno T (1997). Lombard reflex during PAG-induced vocalization in decerebrate cats. Neurosci Res.

[68] Pedersen MB, Egenhardt M, Beedholm K et al. (2024) Superfast Lombard response in free-flying, echolocating bats (conditionally accepted). 10.1016/j.cub.2024.04.048 38744283

[69] Luo J, Kothari NB, Moss CF. (2017) Sensorimotor integration on a rapid time scale. Proceedings of the National Academy of Sciences 114:6605–6610. 10.1073/pnas.1702671114 PMC5488944 28584095

[70] Knörnschild M (2014). Vocal production learning in bats. Curr Opin Neurobiol.

[71] Spoelstra K, van Grunsven RHA, Ramakers JJC, Ferguson KB, Raap T, Donners M, Veenendaal EM, Visser ME. (2017) Response of bats to light with different spectra: light-shy and agile bat presence is affected by white and green, but not red light. Proceedings of the Royal Society B: Biological Sciences 284:20170075. 10.1098/rspb.2017.0075 PMC5454258 28566484

