# Supplementary material for: Daubenton’s bats maintain stereotypical echolocation behaviour and a lombard response during target interception in light
Source: BMC Zool. 2024 Apr 29;9:9. doi: 10.1186/s40850-024-00200-4 (PMC11057132; doi:10.1186/s40850-024-00200-4)
Supplement: Supplementary file 1 — Supplementary Material 1 [file 40850_2024_200_MOESM1_ESM.docx]

**Supplemental material for the research article:**

“Daubenton’s bats maintain stereotypical echolocation behaviour and a Lombard response during target interception in light”

Authors: Astrid Særmark Uebel, Michael Bjerre Pedersen, Kristian Beedholm, Laura Stidsholt, Marie Rosenkjær Skalshøi, Ilias Foskolos and Peter Teglberg Madsen

**Contents:**

**Figures:**

1. Spectra of LED lights 2
2. Error associated with noise removal 2
3. Time to land histogram 3
4. Overview of analysed call data 3
5. Logistic regression of call interval data 4
6. Change in buzz duration and buzz onset range 4
7. Magnitude of SL compensation with range 5

**Tables:**

1. Estimate tables for mixed effect models 6
2. Percentage of trials bats used multiple tries 7
3. p50-values from logistic regression 7
4. Buzz duration 8
5. Buzz onset range 8
6. Magnitude of Lombard response 9

**Figures**


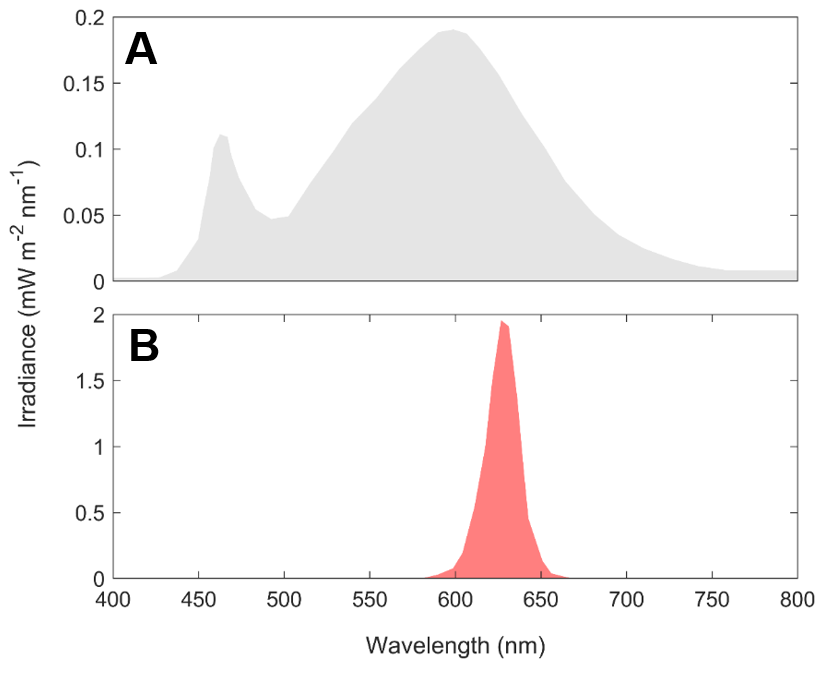


**Figure S1**: Spectra of white (A) and red (B) LED strips.


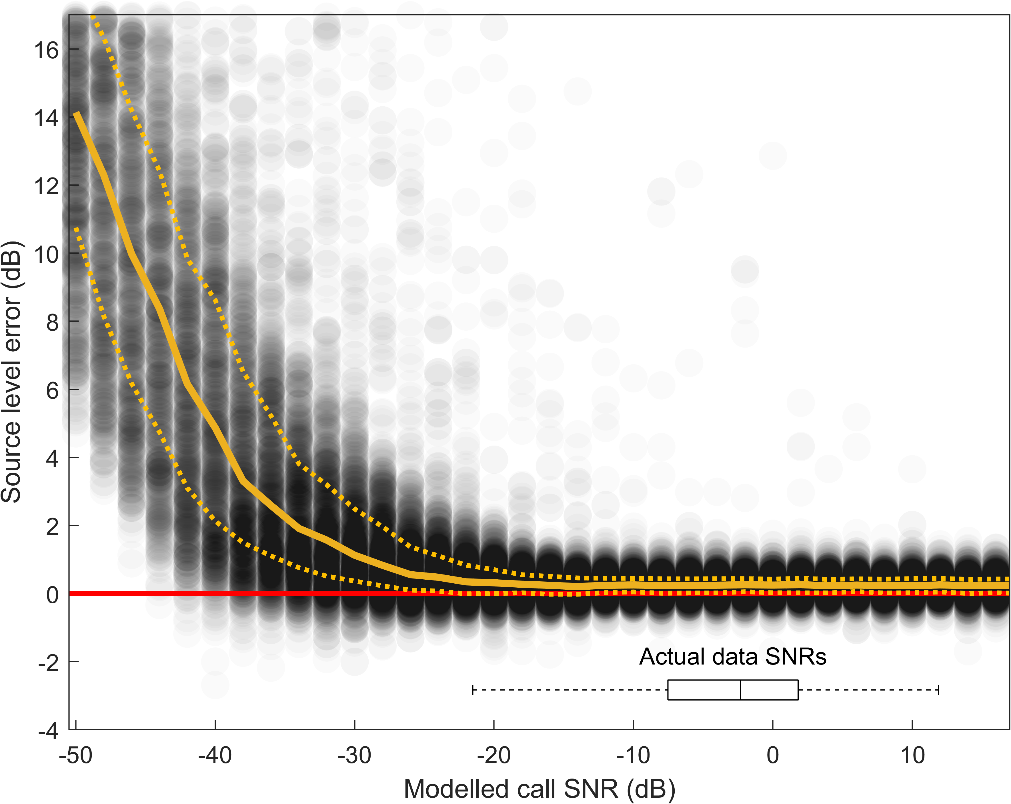


**Figure S2:** Error associated with noise removal. For a random sample of 1000 no-noise calls, noise was modelled and added with different SNRs. SLs were estimated before and after applying our noise removal method. Modelled SNRs are shown on the x-axis and the difference in SL estimation (after noise removal – before noise removal) is shown on the y-axis. The bootstrap shows a median estimation error (solid yellow line) with interquartile ranges (dotted yellow lines). Zero difference (red line) means no error. Positive/negative differences represent overestimation/underestimation of SLs respectively.

**
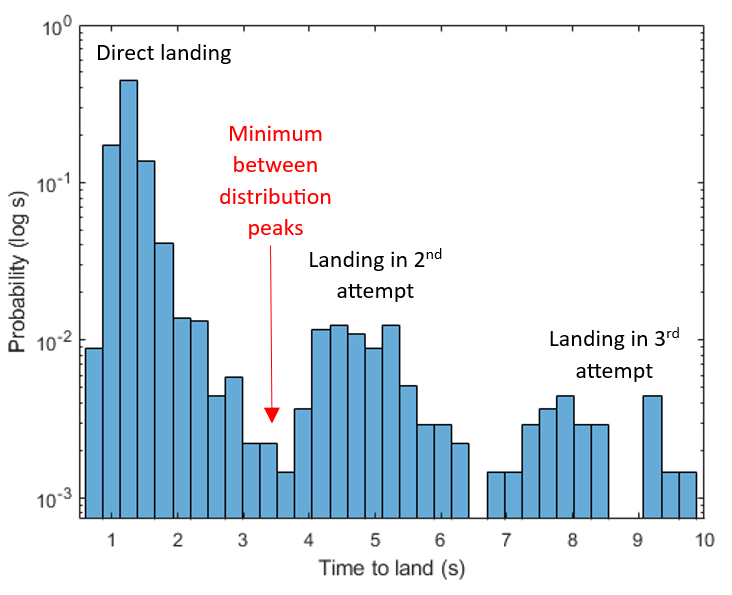
**

**Figure S3:** Histogram of time to land (TTL). One pass corresponds to one round of flight in the flight room without landing on the target.


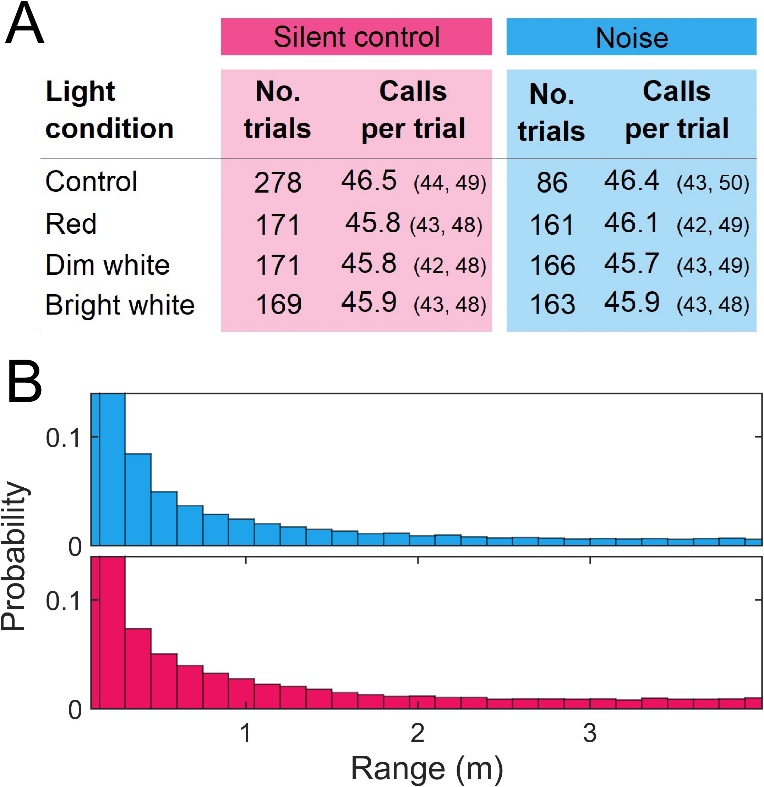


**Figure S4:** Overview of data included in the analysis. A) Number of trials used for call parameter estimation and mean number of calls (inter quantile range) per trial for each treatment. B) Histogram of all call data ranges included in the dataset, showing that localisation was accurate regardless of noise treatment.


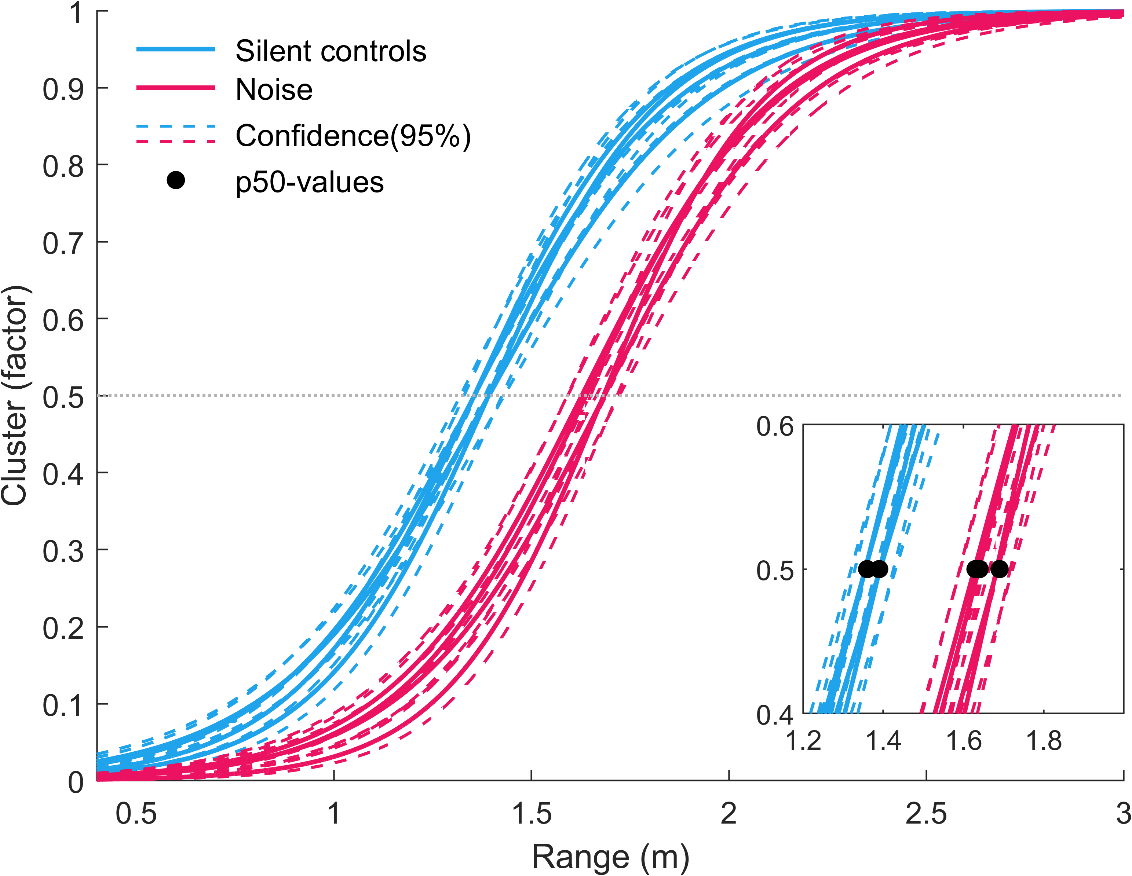


**Figure S5:** Logistic regression of data clusters per light and noise condition. A) Pooled bat data with binomial regression (solid line) and 95% confidence intervals (broken lines). B) Same analysis close-up, with p50 values (black dots). Note: light condition has no effect on adjustments to call rate in silent controls and noise conditions.


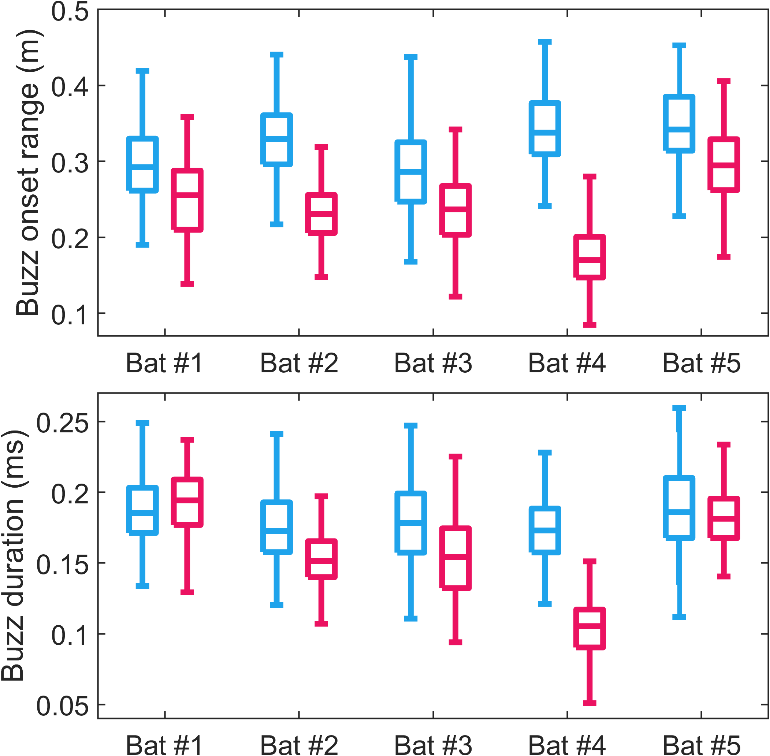


**Figure S6:** Change in buzz duration and buzz onset range per bat ID in silent controls (blue) and noise conditions (magenta).


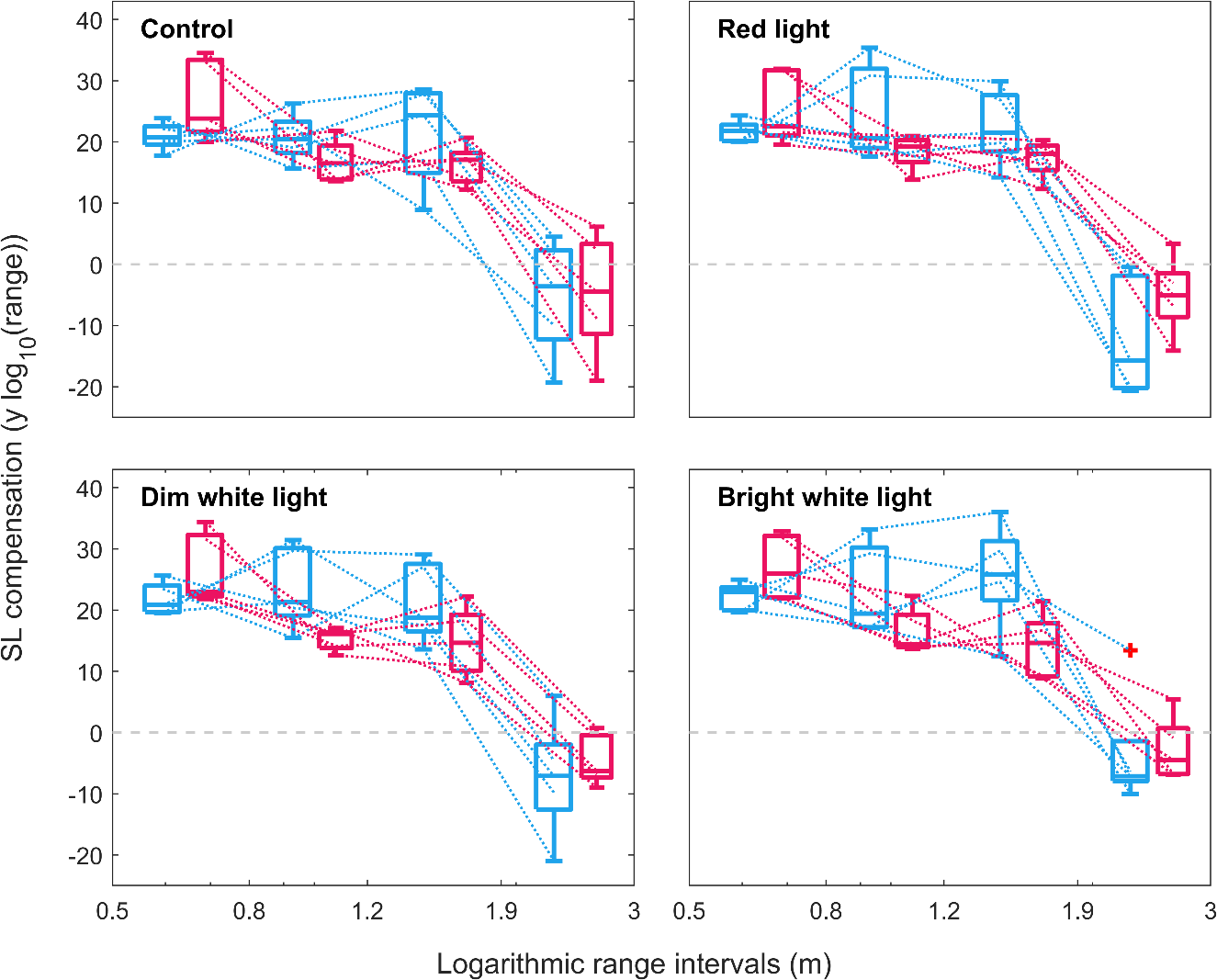


**Figure S7:** Magnitude of the logarithmic SL compensation with range for all light conditions. The magnitude is shown as the slope of the linear regression of SL versus log10(range). Boxplots are given as an average between bats (n = 5) and are computed in logarithmically spaced range bins. Dotted lines connect points from each individual bat. Note: Relative and absolute SL compensation is unchanged across light conditions.

**Tables**

**Table S1:** Estimate tables for Linear Mixed Effect models for time to land, buzz onset range and buzz duration.

| **Model:** Time to land ~ noise condition + light condition + bat ID + (1\|date) | | | | | |
| --- | --- | --- | --- | --- | --- |
| **Name** | **Estimate** | **SE** | **t-value** | **DF** | **p-value** |
| (Intercept) | 1241.11 | 34.60 | 35.88 | 1139 | 3.18E-189 |
| Bat #2 | 147.38 | 25.20 | 5.85 | 1139 | 6.52E-09 |
| Bat #3 | 180.63 | 23.84 | 7.58 | 1139 | 7.32E-14 |
| Bat #4 | -55.90 | 22.94 | -2.44 | 1139 | 1.50E-02 |
| Bat #5 | 103.79 | 28.22 | 3.68 | 1139 | 2.46E-04 |
| Dim white light | -4.82 | 27.91 | -0.17 | 1139 | 8.63E-01 |
| Bright white light | 11.17 | 27.37 | 0.41 | 1139 | 6.83E-01 |
| Red light | 34.34 | 27.66 | 1.24 | 1139 | 2.15E-01 |
| Masking noise | 21.44 | 17.02 | 1.26 | 1139 | 2.08E-01 |

| **Model:** Buzz onset range ~ noise condition + light condition + bat ID + (1\|date) | | | | | |
| --- | --- | --- | --- | --- | --- |
| **Name** | **Estimate** | **SE** | **t-value** | **DF** | **p-value** |
| (Intercept) | 0.32 | 0.00 | 73.63 | 1139 | 0.00E+00 |
| Bat #2 | 0.02 | 0.01 | 4.04 | 1139 | 5.67E-05 |
| Bat #3 | -0.02 | 0.00 | -4.65 | 1139 | 3.74E-06 |
| Bat #4 | 0.00 | 0.00 | -0.15 | 1139 | 8.78E-01 |
| Bat #5 | 0.05 | 0.01 | 8.75 | 1139 | 7.68E-18 |
| Dim white light | -0.01 | 0.00 | -2.61 | 1139 | 9.06E-03 |
| Bright white light | 0.00 | 0.00 | -0.43 | 1139 | 6.65E-01 |
| Red light | 0.00 | 0.00 | -0.85 | 1139 | 3.96E-01 |
| Masking noise | -0.09 | 0.00 | -24.54 | 1139 | 4.54E-107 |

| **Model:** Buzz duration ~ noise condition + light condition + bat ID + (1\|date) | | | | | |
| --- | --- | --- | --- | --- | --- |
| **Name** | **Estimate** | **SE** | **t-value** | **DF** | **p-value** |
| (Intercept) | 0.20 | 0.00 | 84.10 | 1139 | 0.00E+00 |
| Bat #2 | -0.02 | 0.00 | -6.51 | 1139 | 1.12E-10 |
| Bat #3 | -0.02 | 0.00 | -7.88 | 1139 | 7.37E-15 |
| Bat #4 | -0.04 | 0.00 | -15.26 | 1139 | 5.51E-48 |
| Bat #5 | 0.00 | 0.00 | 0.53 | 1139 | 5.98E-01 |
| Dim white light | -0.01 | 0.00 | -2.35 | 1139 | 1.91E-02 |
| Bright white light | 0.00 | 0.00 | -1.60 | 1139 | 1.11E-01 |
| Red light | -0.01 | 0.00 | -2.30 | 1139 | 2.17E-02 |
| Masking noise | -0.03 | 0.00 | -12.86 | 1139 | 2.03E-35 |

**Table S2:**  Percentage (%) of trials where a bat assessed the task multiple times per bat ID, noise condition and light condition.

|  | **Bat #1** | | **Bat #2** | | **Bat #3** | | **Bat #4** | | **Bat #5** | |
| --- | --- | --- | --- | --- | --- | --- | --- | --- | --- | --- |
|  | *Silent* | *Noise* | *Silent* | *Noise* | *Silent* | *Noise* | *Silent* | *Noise* | *Silent* | *Noise* |
| **None** | 5.8 | 10.0 | 6.4 | 40.0 | 11.9 | 19.0 | 3.0 | 31.8 | 14.8 | 22.2 |
| **Red** | 2.6 | 2.9 | 25.8 | 27.6 | 12.8 | 30.6 | 5.3 | 24.3 | 12.5 | 32.0 |
| **Dim white** | 2.7 | 2.7 | 12.5 | 22.6 | 32.4 | 43.2 | 7.7 | 36.1 | 0.0 | 40.0 |
| **Bright white** | 0.0 | 0.0 | 9.7 | 22.6 | 21.6 | 35.1 | 2.7 | 24.3 | 12.0 | 33.3 |

**Table S3:** p50-values and 95% confidence intervals per bat ID, noise condition and light condition. Note: within noise conditions, light has no effect on the onset of call rate adjustments.

|  |  | **Bat #1** | **Bat #2** | **Bat #3** | **Bat #4** | **Bat #5** |
| --- | --- | --- | --- | --- | --- | --- |
| **None** | *Silent* | 0.9  [0.86,0.94] | 1.53  [1.47,1.58] | 1.49  [1.44,1.54] | 1.42  [1.37,1.46] | 1.5  [1.43,1.58] |
|  | *Noise* | 1.64  [1.58,1.74] | 1.4  [1.31,1.53] | 1.7  [1.6,1.82] | 1.82  [1.7,1.91] | 1.63  [1.5,1.87] |
| **Red** | *Silent* | 0.9  [0.84,0.96] | 1.39  [1.33,1.47] | 1.62  [1.53,1.71] | 1.46  [1.38,1.54] | 1.65  [1.55,1.83] |
|  | *Noise* | 1.6  [1.53,1.67] | 1.53  [1.46,1.6] | 1.79  [1.73,1.88] | 1.84  [1.74,1.92] | 1.69  [1.59,1.8] |
| **Dim white** | *Silent* | 0.92  [0.87,0.98] | 1.35  [1.29,1.42] | 1.81  [1.69,1.98] | 1.36  [1.31,1.41] | 1.43  [1.35,1.5] |
|  | *Noise* | 1.64  [1.54,1.7] | 1.51  [1.43,1.57] | 1.77  [1.69,1.85] | 1.78  [1.7,1.85] | 1.45  [1.34,1.55] |
| **Bright white** | *Silent* | 0.98  [0.93,1.03] | 1.44  [1.37,1.51] | 1.64  [1.56,1.71] | 1.42  [1.36,1.48] | 1.52  [1.43,1.6] |
|  | *Noise* | 1.73  [1.66,1.82] | 1.6  [1.53,1.67] | 1.74  [1.69,1.82] | 1.82  [1.75,1.9] | 1.4  [1.35,1.5] |

**Table S4:** Means and 95% quantiles for buzz duration (s) per bat ID, noise condition and light condition.

|  |  | **Bat #1** | **Bat #2** | **Bat #3** | **Bat #4** | **Bat #5** |
| --- | --- | --- | --- | --- | --- | --- |
| **None** | *Silent* | 0.19 (0.15,0.23) | 0.19  (0.13,0.26) | 0.18  (0.12,0.24) | 0.17  (0.13,0.22) | 0.2  (0.16,0.28) |
|  | *Noise* | 0.2  (0.1,0.23) | 0.16  (0.13,0.19) | 0.16  (0.09,0.2) | 0.11  (0.07,0.14) | 0.18  (0.16,0.22) |
| **Red** | *Silent* | 0.18  (0.14,0.24) | 0.17  (0.13,0.23) | 0.17  (0.1,0.23) | 0.18  (0.14,0.23) | 0.18  (0.11,0.22) |
|  | *Noise* | 0.19  (0.14,0.24) | 0.15  (0.11,0.19) | 0.16  (0.11,0.22) | 0.1  (0.05,0.15) | 0.19  (0.16,0.26) |
| **Dim white** | *Silent* | 0.18  (0.15,0.22) | 0.17  (0.11,0.22) | 0.18  (0.13,0.24) | 0.17  (0.12,0.21) | 0.2  (0.16,0.29) |
|  | *Noise* | 0.19  (0.13,0.22) | 0.15  (0.12,0.18) | 0.15  (0.12,0.22) | 0.1  (0.05,0.13) | 0.18  (0.15,0.25) |
| **Bright white** | *Silent* | 0.19  (0.12,0.23) | 0.18  (0.12,0.43) | 0.18  (0.14,0.23) | 0.17  (0.13,0.21) | 0.2  (0.14,0.46) |
|  | *Noise* | 0.19  (0.1,0.23) | 0.15  (0.1,0.19) | 0.16  (0.1,0.22) | 0.11  (0.08,0.15) | 0.18  (0.14,0.23) |

**Table S5:** Means and 95% quantiles for buzz onset range (m) per bat ID, noise condition and light condition.

|  |  | **Bat #1** | **Bat #2** | **Bat #3** | **Bat #4** | **Bat #5** |
| --- | --- | --- | --- | --- | --- | --- |
| **None** | *Silent* | 0.3  (0.22,0.38) | 0.36  (0.28,0.5) | 0.29 (0.2,0.4) | 0.34  (0.26,0.42) | 0.35  (0.28,0.49) |
|  | *Noise* | 0.25  (0.14,0.36) | 0.25  (0.18,0.34) | 0.22  (0.12,0.28) | 0.18  (0.11,0.25) | 0.28  (0.22,0.36) |
| **Red** | *Silent* | 0.29  (0.17,0.4) | 0.31  (0.23,0.38) | 0.28  (0.18,0.39) | 0.36  (0.28,0.45) | 0.36  (0.23,0.45) |
|  | *Noise* | 0.26  (0.18,0.34) | 0.24  (0.17,0.3) | 0.25  (0.17,0.37) | 0.17  (0.1,0.27) | 0.34  (0.25,0.46) |
| **Dim white** | *Silent* | 0.29  (0.23,0.42) | 0.33  (0.22,0.46) | 0.3 (0.2,0.46) | 0.33  (0.25,0.44) | 0.35  (0.26,0.45) |
|  | *Noise* | 0.24  (0.15,0.32) | 0.23  (0.18,0.32) | 0.23  (0.15,0.36) | 0.17  (0.08,0.24) | 0.28  (0.16,0.35) |
| **Bright white** | *Silent* | 0.3  (0.15,0.39) | 0.35  (0.26,0.91) | 0.3  (0.19,0.43) | 0.34  (0.26,0.42) | 0.35  (0.25,0.44) |
|  | *Noise* | 0.25 (0.14,0.35) | 0.23 (0.15,0.32) | 0.26 (0.18,0.41) | 0.18 (0.12,0.27) | 0.29 (0.21,0.36) |

**Table S6:** Mean magnitude of Lombard response (dB/dB_noise_) with 95% quantiles per bat ID and light condition.

|  | **Bat #1** | **Bat #2** | **Bat #3** | **Bat #4** | **Bat #5** |
| --- | --- | --- | --- | --- | --- |
| **None** | 0.21  (0.17,0.25) | 0.17  (0.14,0.21) | 0.15  (0.11,0.19) | 0.18  (0.14,0.21) | 0.22  (0.18,0.26) |
| **Red** | 0.21  (0.17,0.25) | 0.17  (0.14,0.21) | 0.16  (0.12,0.2) | 0.17  (0.14,0.21) | 0.22  (0.18,0.26) |
| **Dim white** | 0.21  (0.16,0.25) | 0.17  (0.13,0.2) | 0.15 (0.11,0.2) | 0.16  (0.12,0.2) | 0.21  (0.17,0.25) |
| **Bright white** | 0.21  (0.17,0.25) | 0.18  (0.14,0.21) | 0.15  (0.11,0.19) | 0.16  (0.13,0.2) | 0.21  (0.17,0.25) |
